# Detecting Microglial Density With Quantitative Multi-Compartment Diffusion MRI

**DOI:** 10.3389/fnins.2019.00081

**Published:** 2019-02-19

**Authors:** Sue Y. Yi, Brian R. Barnett, Maribel Torres-Velázquez, Yuxin Zhang, Samuel A. Hurley, Paul A. Rowley, Diego Hernando, John-Paul J. Yu

**Affiliations:** ^1^Neuroscience Training Program, Wisconsin Institutes for Medical Research, University of Wisconsin–Madison, Madison, WI, United States; ^2^Department of Biomedical Engineering, College of Engineering, University of Wisconsin–Madison, Madison, WI, United States; ^3^Department of Radiology, University of Wisconsin School of Medicine and Public Health, Madison, WI, United States; ^4^Department of Medical Physics, University of Wisconsin School of Medicine and Public Health, Madison, WI, United States; ^5^Department of Psychiatry, University of Wisconsin School of Medicine and Public Health, Madison, WI, United States

**Keywords:** diffusion weighted imaging, NODDI, neuroinflammation, microglia, MRI, DWI, multi-compartment models

## Abstract

Neuroinflammation plays a central role in the neuropathogenesis of a wide-spectrum of neurologic and psychiatric disease, but current neuroimaging methods to detect and characterize neuroinflammation are limited. We explored the sensitivity of quantitative multi-compartment diffusion MRI, and specifically neurite orientation dispersion and density imaging (NODDI), to detect changes in microglial density in the brain. Monte Carlo simulations of water diffusion using a NODDI acquisition scheme were performed to measure changes in a virtual MRI signal following modeled cellular changes within the extra-neurite space. 12-week-old C57BL/6J male mice (*n* = 48; 24 control, 24 treated with colony stimulating factor 1 receptor (CSF1R) inhibitor, PLX5622) were sacrificed at 0, 1, 3, and 7 days following withdrawal of CSF1R inhibition and were imaged *ex-vivo* to obtain measures of the orientation dispersion index (ODI). Following imaging, all brains were immunostained with Iba-1, NeuN, and GFAP for quantitative fluorescence microscopy. Cell populations were calculated with the ImageJ particle analyzer tool; correlation between microglial density and mean ODI values were calculated with Kendall's tau. Monte Carlo simulations demonstrate the sensitivity and positive correlation of ODI to increased occupancy in the extra-neurite space. Commensurate with our simulation data, *ex-vivo* NODDI imaging demonstrates an increase in ODI as microglia repopulate the brain following the withdrawal of CSF1R inhibition. Quantitative immunofluorescence of microglial density reveals that microglial density is positively correlated with ODI and greater hindered diffusion in the extra-neurite space (τ = 0.386, *p* < 0.05). Our results demonstrate that clinically feasible multi-compartment diffusion weighted imaging techniques such as NODDI are sensitive to microglial density and the cellular changes associated with microglial activation and highlights its potential to improve clinical diagnostic accuracy, patient risk stratification, and therapeutic monitoring of neuroinflammation in neurologic and psychiatric disease.

## Introduction

Neuroinflammation plays a critical role in the neuropathogenesis of disorders of the central nervous system (CNS) from ischemic stroke and traumatic brain injury (Iadecola and Anrather, 2011; Woodcock and Morganti-Kossmann, 2013) to Alzheimer's disease, schizophrenia, and major depression (Lull and Block, 2010; Mondelli et al., 2017). Neuroimaging techniques have been developed to characterize neuroinflammatory processes, which generally fall into two methodological categories: positron emission tomography (PET) and MRI (Albrecht et al., 2016). However, despite active research efforts toward PET and MR imaging of neuroinflammation, there remains no routine, widespread, and easily accessible neuroimaging tool available for the study of neuroinflammation.

**Figure 1:**
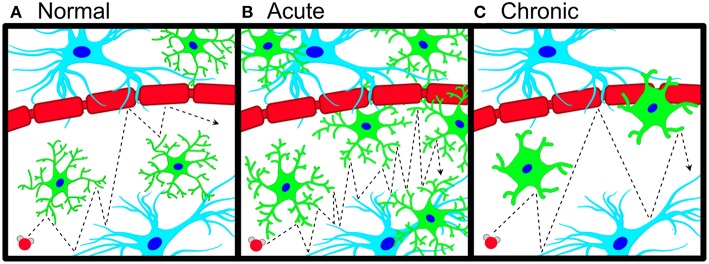
Schematic of microglial neuroinflammation and accompanying changes in water diffusion. **(A)** Pictorial representation of anisotropic water diffusion (red sphere) in the extra-neurite environment in the presence of astrocytes (blue), neuronal/axonal projections (red), and microglia (green). **(B)** During acute neuroinflammation, microglia become hyper-ramified and increase their density in the extra-neurite space, leading to an increase in hindered water diffusion (increased ODI). **(C)** Chronic inflammatory insults cause microglial process to thicken and shorten with a commensurate decrease in microglial density, which leads to decreased occupancy of the extra-neurite space with a decrease in hindered water diffusion (decreased ODI).

Advanced MRI diffusion weighted imaging (DWI) methods represent a conceptually innovative and technically sensitive approach for measuring cellular changes associated with neuroinflammation and microglial activation. Multi-compartment DWI methods such as neurite orientation dispersion and density imaging (NODDI) are designed to measure water diffusion arising from distinct tissue compartments including the extra-neurite compartment (Zhang H. et al., 2012). In the NODDI model, diffusivity in the extra-neurite compartment is measured by ODI (orientation dispersion index). ODI was originally conceptualized to measure how changes in neurite dispersion influence water diffusivity in the extra-neurite space without accounting for the potential contribution that glial cells (such as microglia) can have on quantitative measures of ODI. However, within the extra-neurite compartment reside glial cells, which account for a large percentage of non-neuronal cells in the mouse and human brain (35 and 50%, respectively) (Herculano-Houzel et al., 2006; Azevedo et al., 2009; Herculano-Houzel, 2014; Mota and Herculano-Houzel, 2014; von Bartheld et al., 2016). As microglia comprise 5–15% of all glial cells (Alliot et al., 1999; Ginhoux et al., 2010) and in response to inflammatory stimuli, undergo substantial changes in both morphology and density (Hinwood et al., 2012; Yang et al., 2013), these changes would be expected to significantly alter the degree of hindered diffusion in the extra-neurite compartment. These changes thus offer a potential opportunity to assess microglial activation and microglial-mediated neuroinflammation by probing water diffusion using DWI (Figure 1).

We aimed to characterize the relationship between microglial density and water diffusivity specific to the extra-neurite compartment with multi-compartment diffusion MRI, with a specific focus on NODDI given its clinical feasibility (Rae et al., 2016). While previous work has examined quantitative histological measures of NODDI in the spinal cord (Grussu et al., 2017), the work presented herein is the first to corroborate histological measurements with quantitative measures of diffusion MRI from the extra-neurite space in the brain. We hypothesize that changes in microglial density will alter water diffusivity in the extra-neurite space thus serving as a potential measure of microglial density across a broad spectrum of acute and chronic neuroinflammatory states. To evaluate this hypothesis, we performed Monte Carlo simulations of pulsed gradient measurements of water diffusion, *ex-vivo* NODDI imaging of mice following CSF1R (colony stimulating factor 1 receptor) antagonism, and quantitative histological measurements of microglial density. Together, these data represent a reconceptualization and potential application of multi-compartment diffusion imaging for the sensitive detection of microglial-mediated neuroinflammation.

## Materials and Methods

### Theory and *in silico* Simulation

Multi-compartment diffusion models biophysically model the total DWI signal as a sum of the diffusion weighted signal arising from a combination of biophysical compartments with different underlying cellular microstructures:

(1) $$\begin{array}{r} S=\;S_{0}\;\overset{n}{\underset{i=0}{\sum }}w_{i}S_{i} \end{array}$$

where *S*_0_ is the signal for the non-diffusion weighted (or *b*0) acquisitions, *w*_*i*_ the volume fraction and *S*_*i*_ the signal function for the *ith* of *n* total compartments (Harms et al., 2017). In the NODDI model, the diffusion MRI signal is described as a sum of three non-exchanging biophysical compartments:

(2) $$\begin{array}{r} S=\left(1-v_{iso}\right)\left(v_{ic}S_{ic}+\left(1-v_{ic}\right)S_{ec}\right)+v_{iso}S_{iso} \end{array}$$

where *S* is the entire normalized signal; *S*_*ic*_, *S*_*ec*_, and *S*_*iso*_ are the normalized signals of the intracellular, extracellular, and CSF compartments, respectively, and ν_*ic*_ and ν_*iso*_ are the normalized volume fractions of the intracellular and CSF compartments (Zhang H. et al., 2012).

To test how cellular changes in the extra-neurite space (microglial density) impacts the measured diffusion signal from the extra-neurite space (ODI, orientation dispersion index), an *in silico* diffusion experiment using multiple Monte Carlo random walk simulations as implemented in Camino^1^ (Hall and Alexander, 2009) was performed by varying the number of modeled cells in the extra-neurite space. To generate the components of the multi-compartment diffusion model, basic geometrical components representing white matter axons and microglia were constructed in Blender (Blender Foundation, Amsterdam, Netherlands). We constructed a series of 6 undulating cylinders (with no dispersion) modeling axons in a similar manner as previously described (Kamiya et al., 2017) with radius = 1 μm, length = 40 μm, undulation amplitude *A* = 2, to yield a final λ = 1.024 to simulate a voxel in a white matter tract. Icospheres were next modeled as simplified microglia in the extra-neurite space and were generated with a radius = 5 μm (Kozlowski and Weimer, 2012). The cylinders were then hexagonally packed without touching within the simulated volume (40 × 40 × 40 μm) with all components placed within the model in MatLab (version 2015a, MathWorks, Natick, MA, USA). 10 simulations of 0, 5, 15, and 25 spheres were performed with spheres randomly distributed throughout the extra-neurite space of the modeled volume. The volume fraction of the bundled axons is 2.7%; the volume fraction of the spheres is 6.3%, 18.9%, and 31.5% for 5, 15, and 25 spheres, respectively. Each simulation comprised of 100,000 spins and 5,000 time steps. The free diffusivity was set at 0.6 × 10^−9^ m^2^/s per recommendations in Camino (Cook et al., 2006). From the simulated random walks of particles, a virtual MRI signal was obtained using the NODDI acquisition scheme used in our *ex-vivo* samples with the addition of Gaussian noise to the simulated signal with SNR = 50 of the b = 0 signal for each run. The mean ODI was calculated for each simulation. Diffusion tensor indices of fractional anisotropy (FA) and mean diffusivity (MD) were also calculated.

### Animals and Reagents

All experiments were performed in accordance with animal protocols approved by the Institutional Animal Care and Use Committee at our institution (Protocol #: M005899). 12-week-old C57BL/6J male mice (Charles River Laboratories, MA, USA) were used for all experiments and were randomly assigned to control or experimental CSF1R inhibition cohorts. Control animals were maintained on AIN-76A standard chow (Research Diets, NJ, USA); animals receiving CSF1R inhibition received AIN-76A admixed with the CSF1R inhibitor PLX5622 (Plexxikon, CA, USA; 1,200 mg/kg) as previously described (Elmore et al., 2014). Animals receiving CSF1R inhibition were maintained on their admixed diet for 8-days; on day 8, CSF1R inhibition was withdrawn by replacing their chow with standard chow (AIN-76A). For each time point, mice from the control and the experimental groups were sacrificed on days 0, 9, 11, and 15 (*n* = 48; *n* = 6, each time point; control and experimental).

### MRI Acquisition

#### Data Acquisition

On days 0, 9, 11, and 15, mice were brought to a surgical plane of anesthesia with isoflurane then transcardially perfused with phosphate-buffered solution (PBS) followed by 4% paraformaldehyde (PFA) in 0.1 M PBS. Brains were extracted from the cranial vault and post-fixed in PFA. Imaged brains were placed in a custom-built holder immersed in Fluorinert (FC-3283, 3M, St. Paul, MN, USA) and imaged with a 4.7-T Agilent MRI system with a 3.5-cm diameter quadrature volume RF coil. Multi-slice, diffusion-weighted, spin echo images were used to acquire 10 non-diffusion weighted images (b = 0 s∙mm^−2^) and 75 diffusion-weighted images (25: b = 800 s∙mm^−2^, 50: b = 2,000 s∙mm^−2^), using non-colinear diffusion-weighting directions. Other imaging parameters: TE/TR = 24.17/2000-ms, FOV = 30 × 30 mm^2^, matrix = 192 × 192 reconstructed to 256 × 256 for an isotropic voxel size of 0.25-mm over two signal averages. All animals were used in subsequent analyses.

#### Data Preprocessing and Region of Interest (ROI) Analysis

Raw data files were converted to NIfTI format and FSL was used to correct for eddy current artifacts with Eddy-correct. FSL output volumes were converted to NIfTI tensor format for use with the DTI-TK software package. DTI-TK (Zhang et al., 2006) was used to estimate a study-specific tensor template, to which subject tensor volumes were spatially normalized. The NODDI model was then voxel-wise fitted to the diffusion data in Matlab (The MathWorks, Inc., Natick, MA) with the NODDI toolbox^2^. An additional compartment of isotropic restriction was employed for *ex-vivo* studies as recommended (Alexander et al., 2010). A manual ROI was drawn over the left dentate gyrus from anatomically defined areas on a normalized mean diffusion map. The ROI was overlaid over subjects from each of the two groups (± CSF1R treatment) and ODI, FA, and MD were calculated.

### Immunofluorescent Staining and Quantification

Following imaging, brains were removed from their custom holders and were returned to ice-cold 4% PFA for 24 h, then in a 30% sucrose solution (Alfa Aesar, Ward Hill, MA; Cat# 36508) in 0.1 M PBS (Growcells, Irvine, CA; Cat# MRGF-6235). Frozen coronal sections were taken at 40 μm using a cryostat (Leica CM 1850, Wetzlar, Germany) and stored short-term in PBS at 4°C until staining. Floating sections were incubated in blocking solution formulated with 0.1 M PBS, 2% bovine serum albumin (Fisher Scientific, Hampton, NH; Cat# BP9706-100) and 0.1% sodium azide (Sigma, St. Louis, MO; Cat# S2002) for 1 h at room temperature (RT), then incubated overnight at 4C with primary antibodies for Iba-1 (rabbit Anti-Iba-1, dilution 1:2000, Abcam, Cambridge, MA, Cat # AB178847), NeuN (chicken Anti-NeuN, dilution 1:1500; EMD Millipore, Billerica, MA Cat# ABN91MI), and GFAP (mouse Anti-GFAP, dilution 1:1000; Thermo Fisher Scientific, Waltham, MA Cat# PIMA512023). Sections were incubated for 1 h at RT with the corresponding Alexa 488-, 555-, 647-labeled species specific secondary antibodies (goat anti-rabbit, Abcam, Cambridge, MA, Cat# AB150077; goat anti-chicken, Thermo Fisher Scientific, Waltham, MA former Invitrogen Cat# A-21437; goat anti-mouse, Abcam, Cambridge, MA, Cat# AB150115; all diluted at 1:2000). Sections were counterstained with 0.1 μm/mL 4',6-diamidino-2-phenylindole (DAPI) (Novus Biologicals, Littleton, CO; Cat# NBP2-31156) for 5 min at RT, then mounted with Fluoromount-G (Southern Biotech, Birmingham, AL, Cat# 0100-01). Images of the left hippocampus were acquired with a Leica DMi8 Inverted Fluorescent microscope (Wetzlar, Germany) with a 10x dry objective lens. All microscopy images were analyzed using ImageJ. The Region of Interest (ROI) manager tool was used to isolate the hippocampus. Images were made binary via manual thresholding, then the Particle Analyzer tool was used to automatically count cells.

### Statistical Analysis

Imaging sample sizes and power analyses are based on standard deviations from previous studies with a significance level of 5% and power of 90% (Ong et al., 2018). Statistical tests were performed in GraphPad Prism or R. Analysis of cell counts between control and CSF1R-inhibitor diet were performed using a two-tailed unpaired Student's *t*-test; *p* < 0.05 was established as the significance level. Kendall's tau coefficient was calculated to measure the non-parametric, ordinal association between microglial cell counts and mean ODI from three time-points in CSF1R administered animals.

## Results

### Computational Modeling of the Extra-Neurite Space in Multi-Compartment MRI

**Figure 2:**
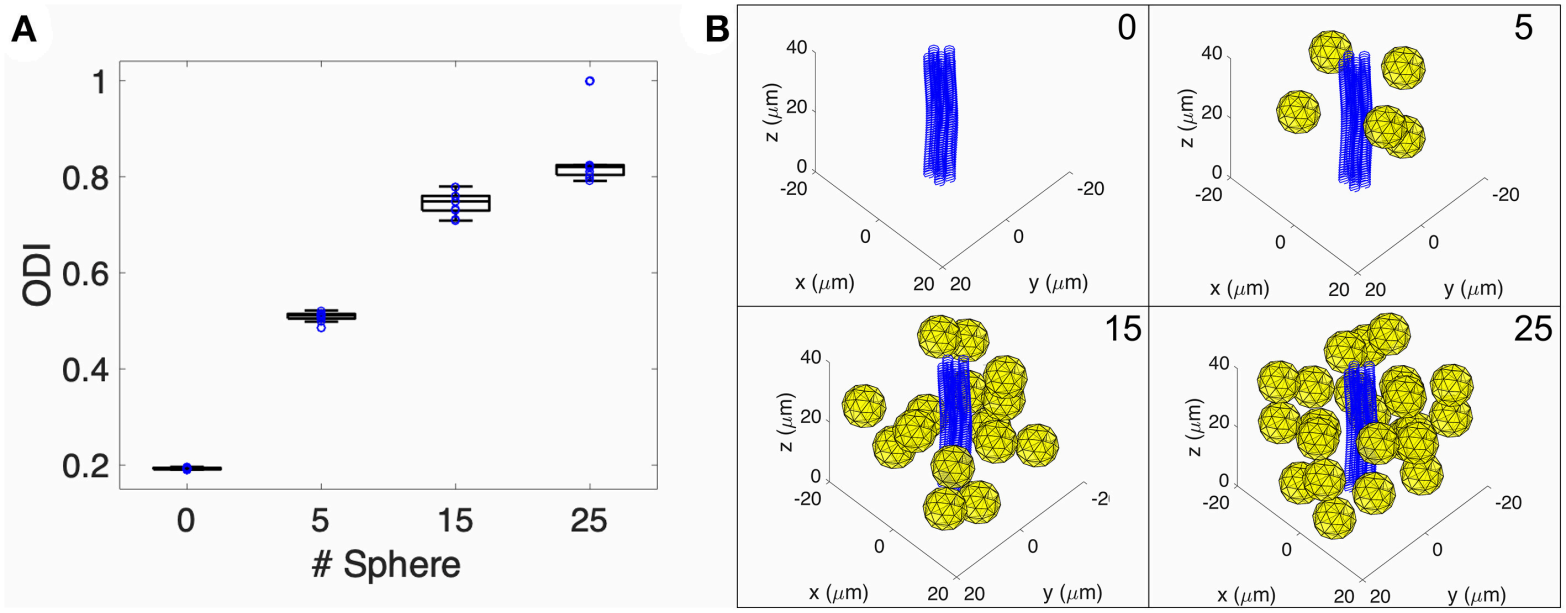
Monte-Carlo NODDI diffusion MRI simulation. **(A)** Box plot of pulsed gradient water diffusion simulations within a representative voxel were performed with 0, 5, 15, and 25 spheres present (representing extra-neurite cellular elements) demonstrating increased ODI as a function of increased occupancy of the extra-neurite space. **(B)** Pictorial representation of the geometry within a single voxel in the Monte-Carlo simulation with blue tubes representing an axon bundle and yellow spheres representing microglia.

As the NODDI model includes parameters to measure water diffusion in the extra-neurite space, we hypothesized that changes in microglial density would change the water diffusivity measured within the extra-neurite compartment. To test this hypothesis and to first ascertain the sensitivity of the extra-neurite compartment to the cellular changes of neuroinflammation, we performed an *in silico* diffusion experiment utilizing a Monte Carlo random walk simulation with NODDI acquisition parameters (Figure 2, Supplementary Figures 1, 2). Within a simulated voxel with a modeled undulating axon bundle (to replicate a white matter tract) (Kamiya et al., 2017), we varied the number of modeled microglia within the simulated voxel over multiple iterative simulations to assess the sensitivity of NODDI to these microglial changes in the extra-neurite space expected during neuroinflammation. FA and MD were also calculated (Supplementary Figure 3). As shown in Figure 2, an increase in the number of microglia accompanies a concomitant increase in ODI, demonstrating that increased occupancy within the extra-neurite space is coupled with increased hindered water diffusion. Our simulation of a voxel in a white matter tract also importantly finds that measures of ODI are independent of neurite dispersion, for which ODI was originally modeled to measure. In Monte Carlo simulations with only the axon bundle present (no microglia), our simulations return a non-zero value of ODI, supporting the hypothesis that any structure localizing to the extra-neurite space (such as the modeled axon bundle) is able to contribute to alterations in water diffusivity within the extra-neurite compartment and thus to calculated values of ODI.

### Quantitative Diffusion MRI of the Extra-Neurite Space Is Sensitive to Microglial Density

**Figure 3:**
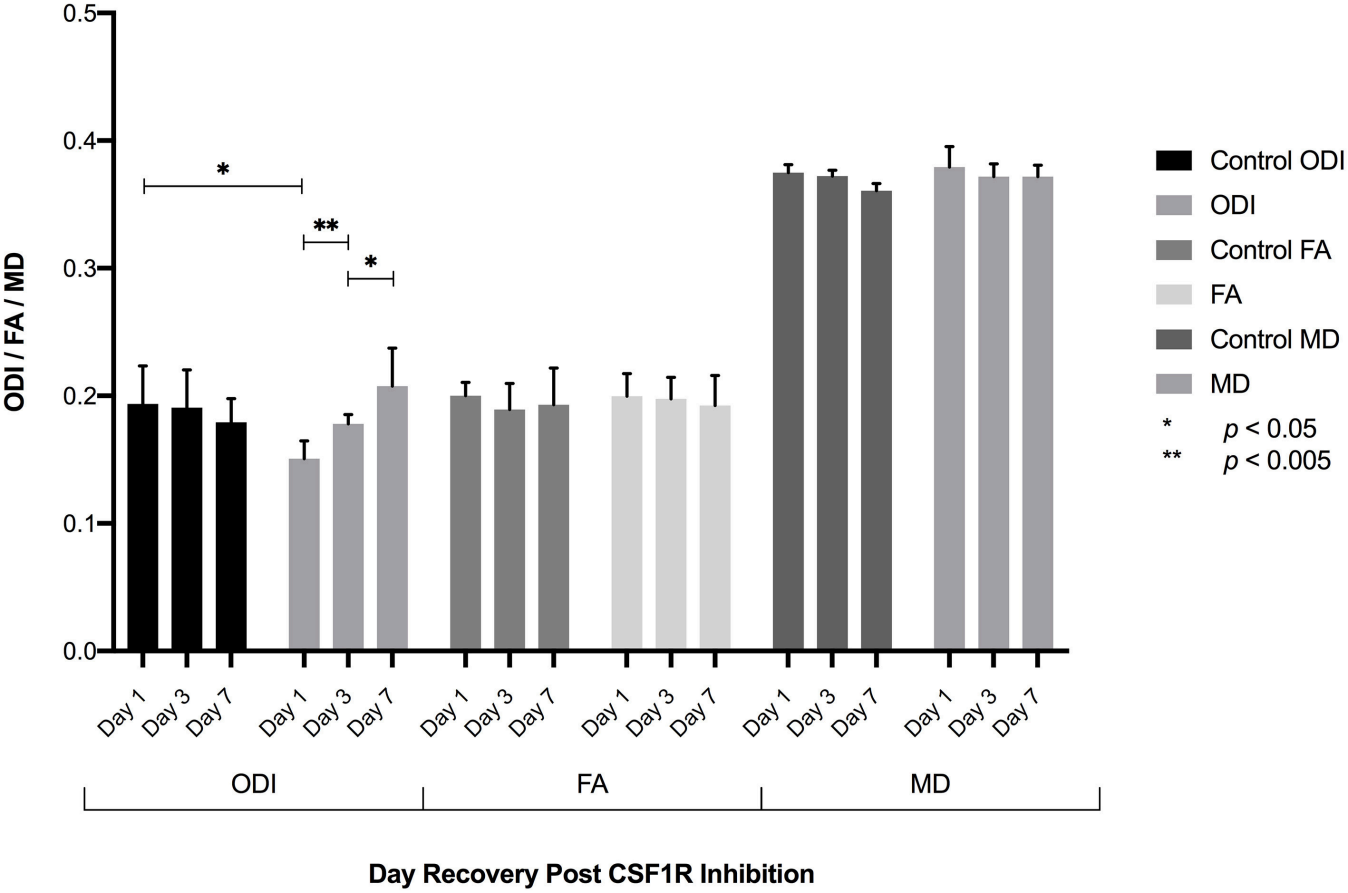
Following the elimination of microglia, CSF1R inhibition was withdrawn allowing microglia to repopulate the brain. ROI analysis of the dentate gyrus 1, 3, and 7 days following inhibitor withdrawal demonstrate an increase in ODI as microglia repopulate the brain with statistically significant differences in ODI between control and day 1 animals, day 1-day 3, and day 3-day 7 animals. No significant difference in ODI is observed between control and D7 animals, consistent with fully repopulated microglial populations in the brain. No significant differences in FA or MD were found.

The extra-neurite compartment includes microglia and other cell populations including astrocytes, oligodendrocytes, ependymal cells, and vascular structures, all of which could be expected to impact the degree of hindered diffusion in the extra-neurite space. To examine the contribution of microglia to the measured diffusion tensor arising from the extra-neurite compartment in the NODDI model, we selectively eliminated microglia from the brain via CSF1R inhibition to specifically characterize the relationship between quantitative measures of ODI and microglial density (Elmore et al., 2014). Following the complete elimination of microglia from the brain following CSF1R inhibition, CSF1R inhibition was withdrawn and NODDI imaging of the dentate gyrus of the hippocampus was performed 1, 3, and 7 days after inhibitor withdrawal (Elmore et al., 2014). At day 1 post-withdrawal during which few microglia are present, we find a statistically significant decrease in ODI when compared to control animals (no CSF1R inhibition) consistent with results derived from our *in silico* model (Figure 3, Supplementary Figure 4). As microglia begin to repopulate the brain following the cessation of CSF1R inhibition, there is an increase in ODI on days 3 and 7, consistent with our *in silico* model's prediction, and further supports both the role of microglia and their contribution to water diffusivity in the extra-neurite space as well as the overall sensitivity of NODDI to capture the cellular changes in microglial density throughout the extra-neurite space (Figure 3). No statistically significant changes in FA or MD were found.

### Microglial Density Is Strongly Correlated With ODI

**Figure 4:**
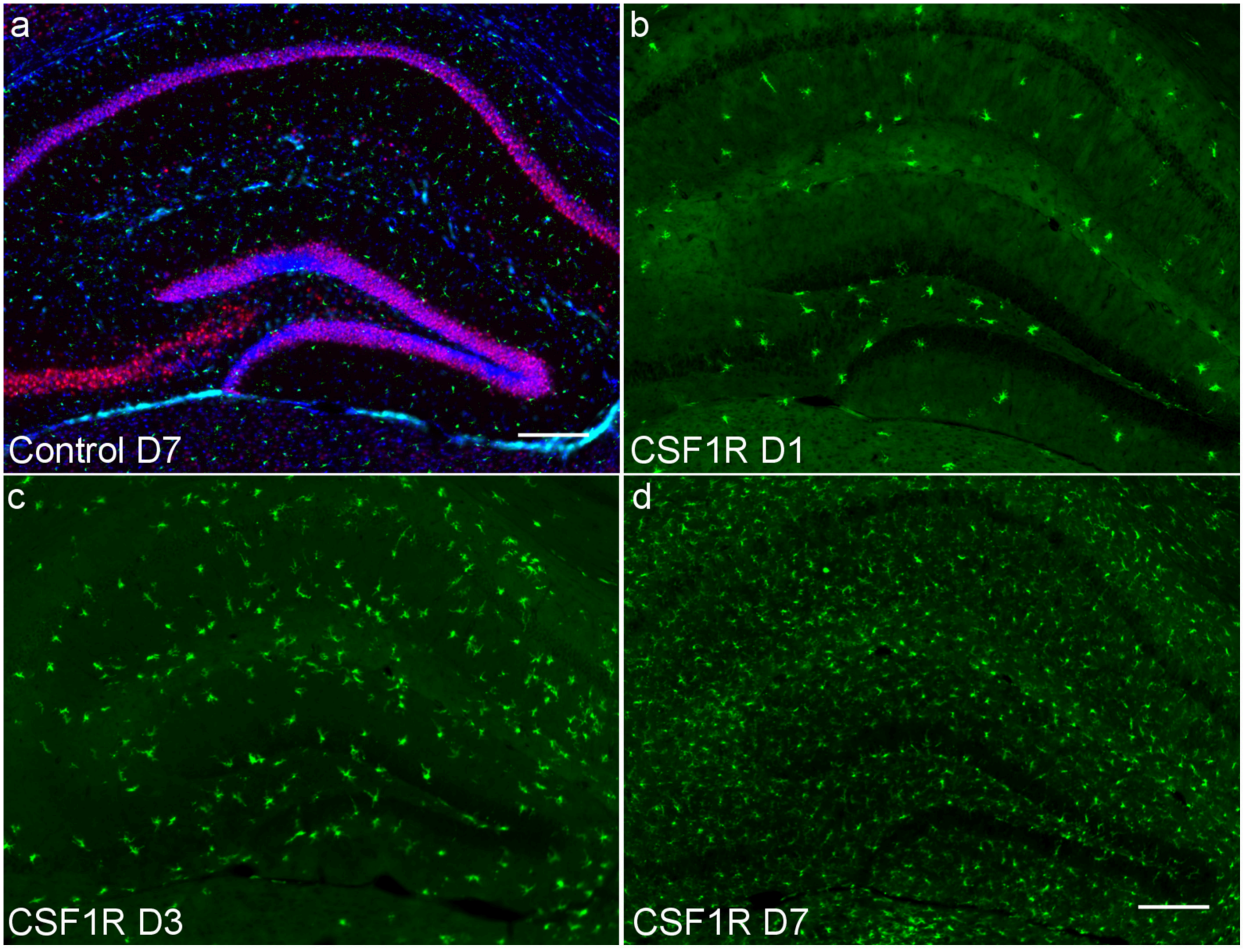
Hippocampal photomicrographs (10X) representative of C57BL/6J mice during microglial repopulation. **(a)** Representative control animal immunostained with antibodies for neurons (anti-NeuN, red), microglia (anti-Iba1, green), and astrocytes (anti-GFAP, cyan), counterstained for nuclei with DAPI (blue). **(b)** Day 1 post CSF1R inhibition display scant microglia present as microglia begin to start repopulating the brain. **(c)** 3 days post-withdrawal and **(d)** 7 days post-withdrawal show microglial recovery over the span of a week. Only microglial counts show a significant increase during the time course. Scale bar = 200 μm.

**Figure 5:**
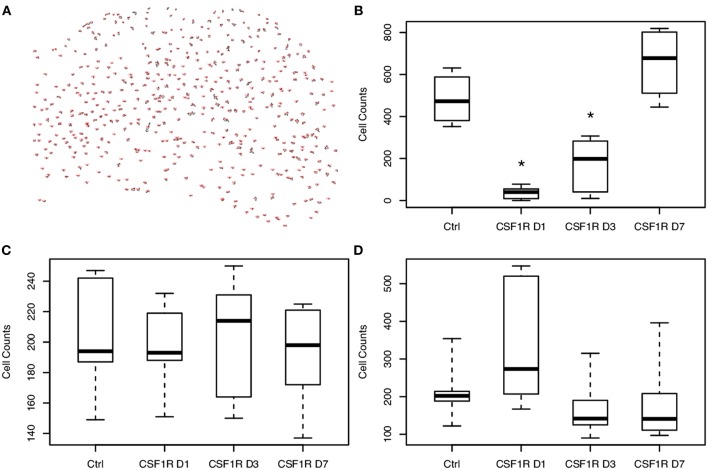
Analysis of cellular density show temporally-dependent increase in microglial density following CSF1R inhibitor withdrawal. **(A)** Representative image of microglial counts from the hippocampus of a control animal produced by ImageJ. Cells were counted following thresholding with the particle analyzer tool. **(B)** Microglia are depleted with CSF1R inhibition and begin to repopulate the brain following CSF1R inhibitor withdrawal. On days 1 and 3 post-withdrawal, microglial counts are still significantly reduced compared to control (^*^p < 0.05). Neurons **(C)** and astrocytes **(D)** demonstrate no significant change in density throughout CSF1R inhibitor treatment or withdrawal.

To further establish whether the measured increase in mean ODI correlates with changes in microglial density, sections of the imaged brains at 1, 3, and 7 days following CSF1R inhibition were stained with Iba1, NeuN, and GFAP to identify microglia, neurons, and astrocytes, respectively. Stained and quantified sections were taken at the level of the hippocampal head that were to co-registered to mean FA maps. Immunofluorescent (IF) staining showed successful microglial depletion following 8 days of CSF1R inhibition with further IF quantification demonstrating no significant difference in neurons or astrocytes (data not shown), recapitulating data previously shown by Elmore et al. (2014). At 1, 3, and 7 days following withdrawal of CSF1R inhibition, there is a steady repopulation of microglia throughout the dentate gyrus (Figure 4), again with no significant change in other major cells populations present in the extra-neurite space (Figure 5, Supplementary Figure 5).

**Figure 6:**
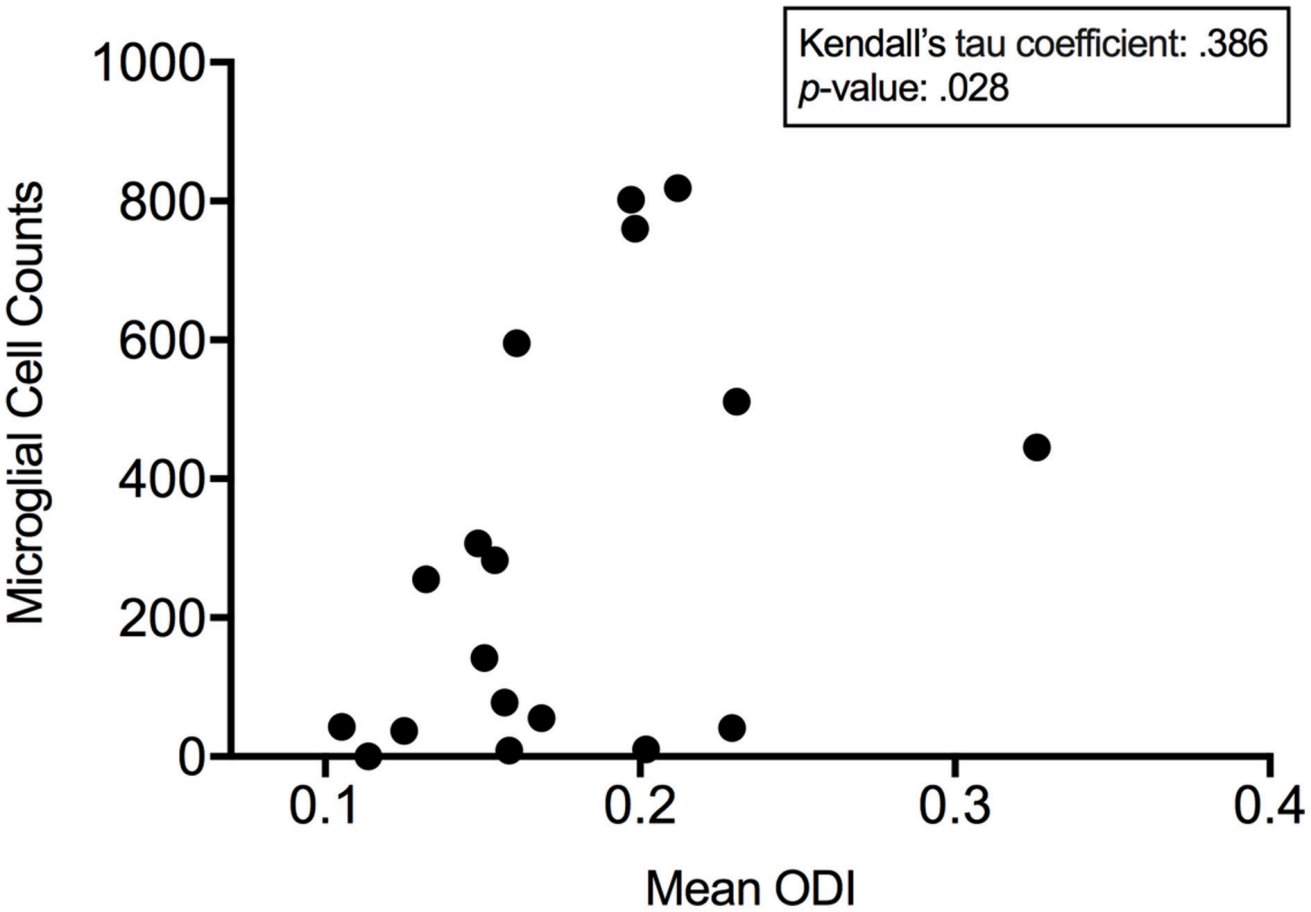
ODI is positively correlated to microglial density. Kendall's tau demonstrates a significant association between measured microglial cell counts and mean orientation dispersion index 1, 3, and 7 days post CSF1R inhibition demonstrating that microglial density is positively correlated with quantitative measures of anisotropic diffusion arising from the extra-neurite space.

With ODI values and quantitative IF data for the number of microglia present, a Kendall's tau coefficient was calculated to measure the non-parametric, ordinal association between microglial cell counts and mean ODI from these three time-points in CS1R administered animals. With a Kendall's tau of 0.386 (*p* = 0.028), we demonstrate that there is a significant association between measured values of ODI and microglial density (Figure 6). These results also align with our *in silico* analysis and show that microglial density is positively correlated with quantitative measures of greater hindered diffusion arising from the extra-neurite space.

## Discussion

The development of DWI and subsequent introduction of diffusion tensor imaging (DTI) have demonstrated water molecules diffuse differently in tissues depending on their type, integrity, and architecture (Soares et al., 2013) making diffusion imaging a promising tool for studying the microstructure of the brain. As an extension of DTI, more sophisticated diffusion imaging techniques as CHARMED (Assaf and Basser, 2005), AxCaliber (Assaf et al., 2008), and NODDI (Zhang H. et al., 2012) model water diffusion in distinct compartments in the brain (intra-neurite, extra-neurite) and provide greater tissue specificity than DWI/DTI. Of these, NODDI represents the first clinically feasible multi-compartment DWI method owing to the prohibitive scan times and the complexity of analyzing data in other multi-compartment methods (Van Hecke et al., 2016). As with the other multi-compartment DWI models, the NODDI model includes terms to measure water diffusion arising from the extra-neurite compartment. With microglia in the extra-neurite compartment undergoing dynamic changes in density and morphology throughout all stages of neuroinflammation (Yang et al., 2013), we hypothesized that these changes were likely to disrupt and alter water diffusivity in the extra-neurite compartment thus raising the possibility of employing multi-compartment DWI for the sensitive detection of the density changes associated with microglial-mediated neuroinflammation.

In this work, we first demonstrate the sensitivity of the NODDI model to capture changes in microglial density, whereby increased occupancy of the extra-neurite space is correlated with greater hindered diffusion. We also show that NODDI is sensitive to microglial density following microglial depletion with CSF1R inhibition and subsequent repopulation after drug removal, revealing that microglial density is a key contributor to quantitative measures of hindered diffusion in the extra-neurite space. Finally, we demonstrate the significant statistical correlation between microglial density with quantitative measures of ODI, showing that microglial density is positively correlated with hindered diffusion in the extra-neurite space. Together these data provide the first example of MRI to track the cellular changes associated with microglial activation during neuroinflammation.

The ability to track microglial activation via changes in microglial density throughout stages of neuroinflammation (Dheen et al., 2007) suggests an exciting potential for NODDI to be a major advance in clinical care and research across a large spectrum of neurologic and psychiatric disease, particularly in clinical diagnostic accuracy, patient risk stratification, and therapeutic monitoring of neuroinflammation. Previous work has examined the impact of peripheral inflammation on NODDI metrics of NDI and ODI (Dowell et al., 2018) and interestingly demonstrate that while no changes in NDI or ODI were found following the administration of interferon-α (IFN-α) the changes in NDI observed, however, could predict the development of long-term fatigue in a subset of patients. These findings highlight the potential of sensitive quantitative multi-compartment diffusion methods in diagnosis and monitoring of neuropsychiatric disease. As a parallel to tracking disease progression, NODDI may also provide a useful neuroimaging biomarker for evaluating the efficacy of new therapeutics. In diseases like Alzheimer's disease (AD), where neuroinflammation is recognized as a key driving force of disease progression (Readhead et al., 2018), therapeutic research is shifting toward targets that may help control the inflammatory response (Ferretti et al., 2012). Clinical evaluation of AD is difficult and relies heavily on observation of symptoms. Although PET has been proposed as a potential method of monitoring AD progression as well as responsivity to anti-inflammatory therapies (Jack et al., 2013), PET methods such as TSPO (translocator protein) imaging harbor a number of limitations including genotypic variation, complex tracer kinetics, and variability of plasma free fractions across human clinical cohorts (Turkheimer et al., 2015). Further studies evaluating use of NODDI vis-à-vis to TSPO imaging in models of neuroinflammation will clarify which imaging modality may have greater sensitivity and clinical viability.

Importantly, we acknowledge that the original formulation of NODDI does not fully account for the biological observations seen in the data presented herein and cautiously temper the translation of this approach in *in vivo* applications (both preclinical and clinical). ODI, as derived from a Watson distribution of stick functions with terms for extracellular diffusion (Zhang H. et al., 2012), was not designed to capture changes in microglial density. Despite this limitation, it is readily apparent that the model is responsive to biophysical changes associated with microglial density to yield new insights into the organization of brain tissue in both health and disease. Results from our simulation experiment should also be interpreted with caution as the simulated voxel size is small and large voxels are known to generate somewhat more realistic dMRI signals (Romascano et al., 2018). Further to this point and potentially also limiting the simulation data are that our *in-silica* model of white matter is not a realistic model of white matter with a low volume of intra-axonal space (2.7%). Additionally, we acknowledge that PFA fixation can subtly alter tissue microstructure and diffusion MR measurements (Zhang J. et al., 2012). Nevertheless, *ex-vivo* imaging is pursued herein as higher SNR and spatial resolution are made possible by longer scan times thereby leading to increased imaging sensitivity and to additionally allow for direct radiologic-pathologic comparisons between our histological and imaging measurements, obviating potential discrepancies that could potentially arise if we were to compare quantitative *in vivo* diffusion measurements and *ex vivo* histopathology. Another potential limitation of our work is that while we have demonstrated the robust sensitivity of measures of ODI to changes in microglial density, other cellular changes taking place in the extra-neurite compartment could have a similar effect on ODI and limit the broad application of our approach. In particular, changes such as the regional breakdown of the blood-brain barrier permitting the infiltration of peripherally circulating lymphocytes and monocytes into the brain parenchyma (as could be seen in the setting of tumors or ischemia), could lead to non-specific findings and would limit the clinical translation of our approach. Although NODDI may not specifically track changes in microglia, this work demonstrates the ability of diffusion weighted imaging to track cellular changes in the brain. Furthermore, these potential shortcomings can be averted with appropriate patient selection (e.g., exclusion of patients with brain tumors or large territory stroke) coupled with future technical development to address issues of specificity. Comparing the performance of other multi-compartment models (e.g., CHARMED, AxCaliber) to NODDI would also contribute to validating the application of multi-compartment diffusion models for the sensitive detection of microglial activation in neuroinflammation, but might be of limited clinical benefit due to acquisition scan times that are outside of potential clinical translation.

In summary, our results demonstrate that NODDI parameters corresponding to the extra-neurite compartment can sensitively detect a broad range of microglial densities in the extra-neurite compartment. With microglial density serving as an important biomarker of disease activity and chronicity across a broad-spectrum of neurologic and psychiatric disease, our results highlight the potential for NODDI and other multi-compartment diffusion MRI techniques to detect the cellular changes of microglial-mediated neuroinflammation toward improving clinical diagnostic accuracy, patient risk stratification, and therapeutic monitoring.

## Author Contributions

SY and JY wrote the manuscript. SY, BB, MT, YZ, SH, and PR performed all experimental work. DH and JY interpreted the experimental data. JY is the guarantor of this work, and as such, had full access to all the data in the study and assumes responsibility for the integrity of the data and the accuracy of the data analyses.

### Conflict of Interest Statement

The authors declare that the research was conducted in the absence of any commercial or financial relationships that could be construed as a potential conflict of interest.

## Footnotes

^1^http://camino.cs.ucl.ac.uk/

^2^http://nitrc.org/projects/noddi_toolbox
