# Supplementary material for: Detecting Microglial Density With Quantitative Multi-Compartment Diffusion MRI
Source: Front Neurosci. 2019 Feb 19;13:81. doi: 10.3389/fnins.2019.00081 (PMC6389825; doi:10.3389/fnins.2019.00081)
Supplement: Supplementary file 1 [file Data_Sheet_1.docx]

Supplementary Material

Detecting Microglial Density with Quantitative Multi-compartment Diffusion MRI

Sue Y. Yi, Brian R. Barnett, Maribel Torres-Velázquez, Yuxin Zhang, Samuel A. Hurley, Paul A. Rowley, Diego Hernando, John-Paul J. Yu^*^

*** Correspondence:** John-Paul J. Yu: [jpyu@uwhealth.org](mailto:jpyu@uwhealth.org)

**
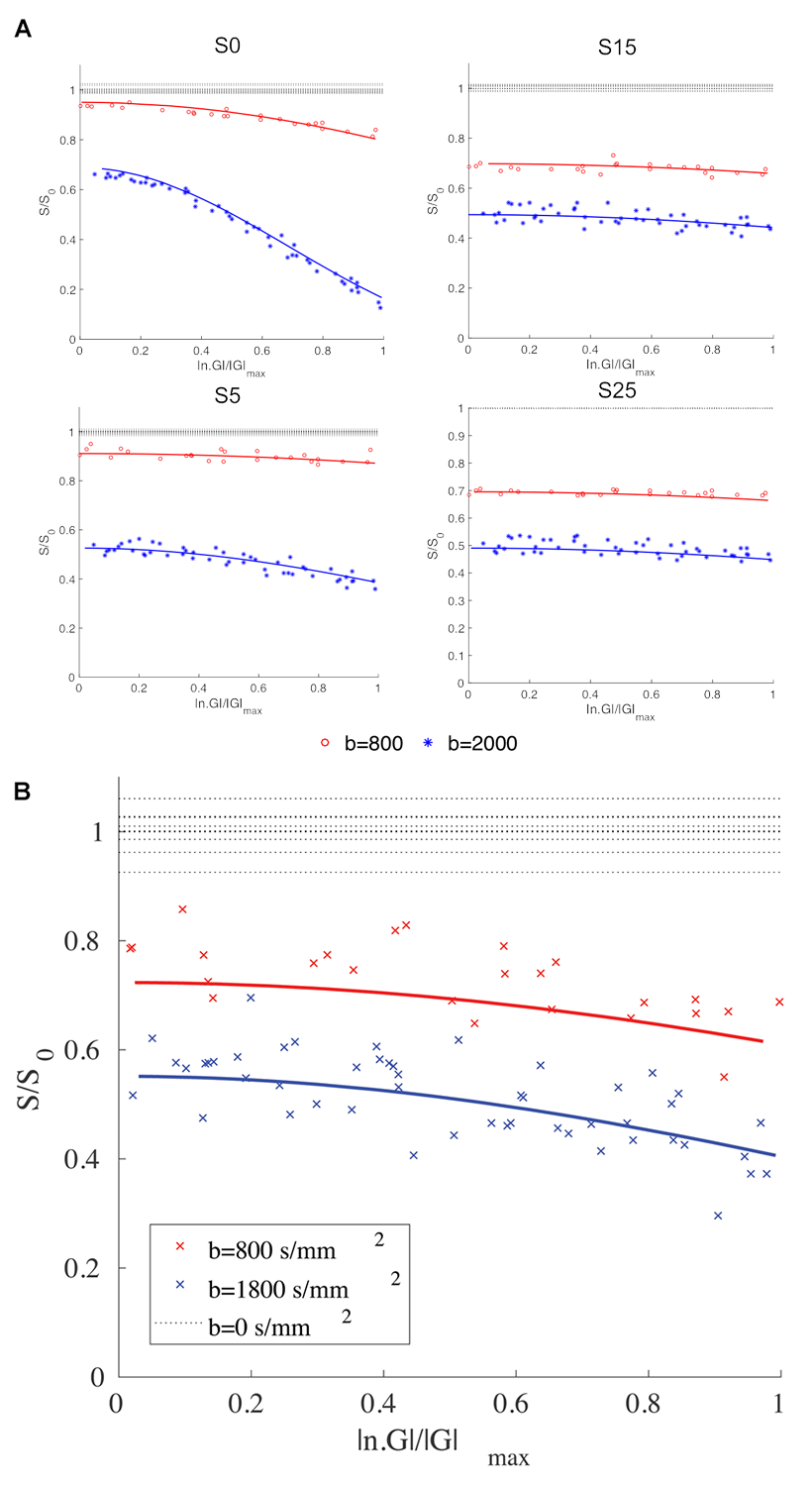
**

**Supplementary Figure 1: (A)** Plots of normalized synthetic signals from simulation data with 0, 5, 15, and 25 spheres for b=800s/mm^2^ and b=2000s/mm^2^. **(B)** Plot of the measurement of a voxel in the corpus callosum in a representative animal on day 1 post-CSF1R inhibition. All measurements are normalized by the single estimate of S^*^_0_ from the NODDI model fitting. The solid lines show the predicted signals from the fitted model.

**
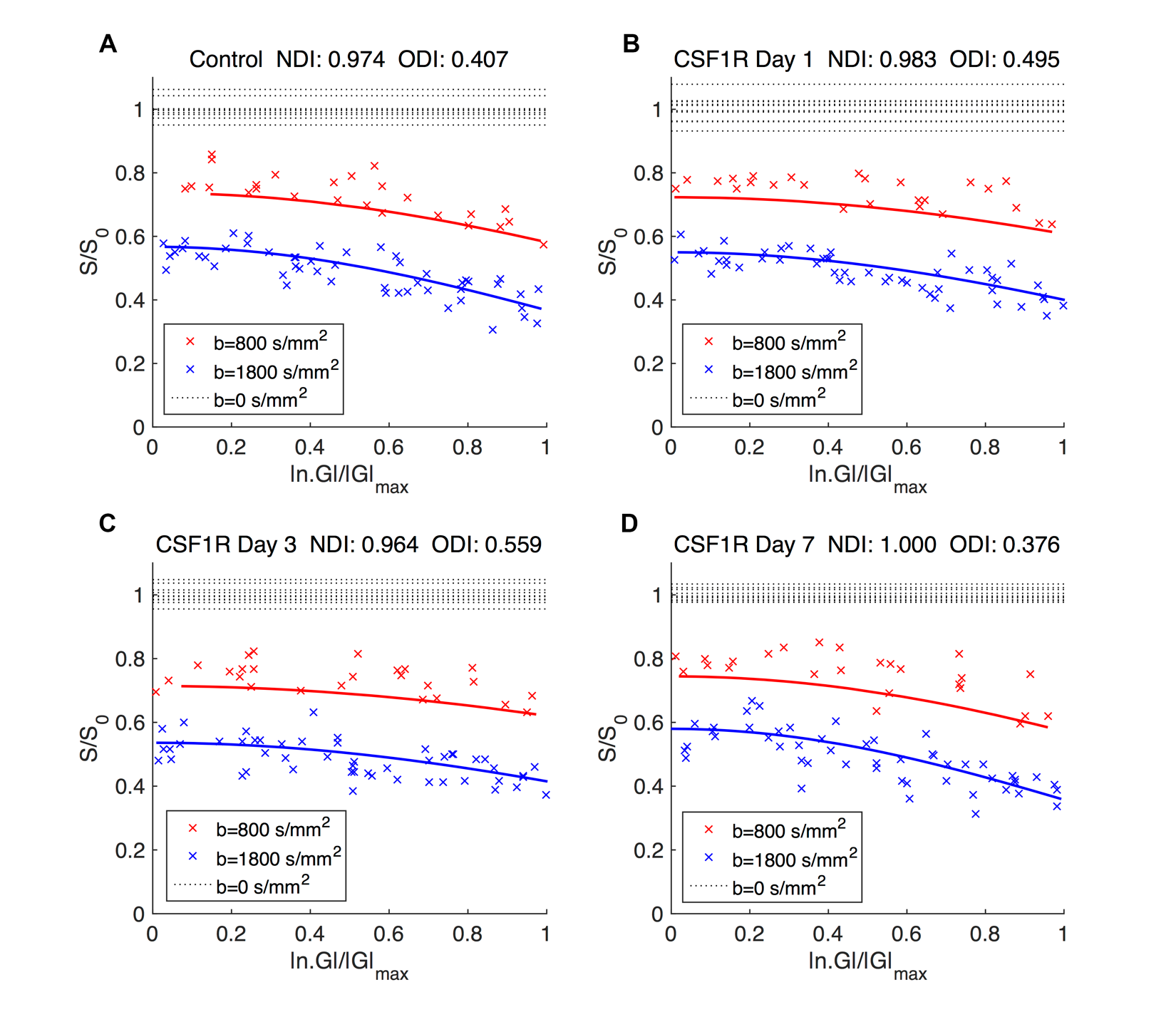
Supplementary Figure 2:** Plots of the measurement of a voxel in the left dentate gyrus of a control animal **(A)** and on days 1, 3, and 7 post-CSF1R inhibition **(B-D)** with voxel-specific calculated NODDI metrics for reference. All measurements are normalized by the single estimate of S^*^_0_ from the NODDI model fitting. The solid lines show the predicted signals from the fitted model.

**Supplementary Figure 3:** Calculated FA and MD (10^-3^mm^2^/s) values for the performed *in silico* diffusion experiment using multiple Monte Carlo random walk simulations as implemented in Camino for different number of spheres occupying the extra-neurite space.

**
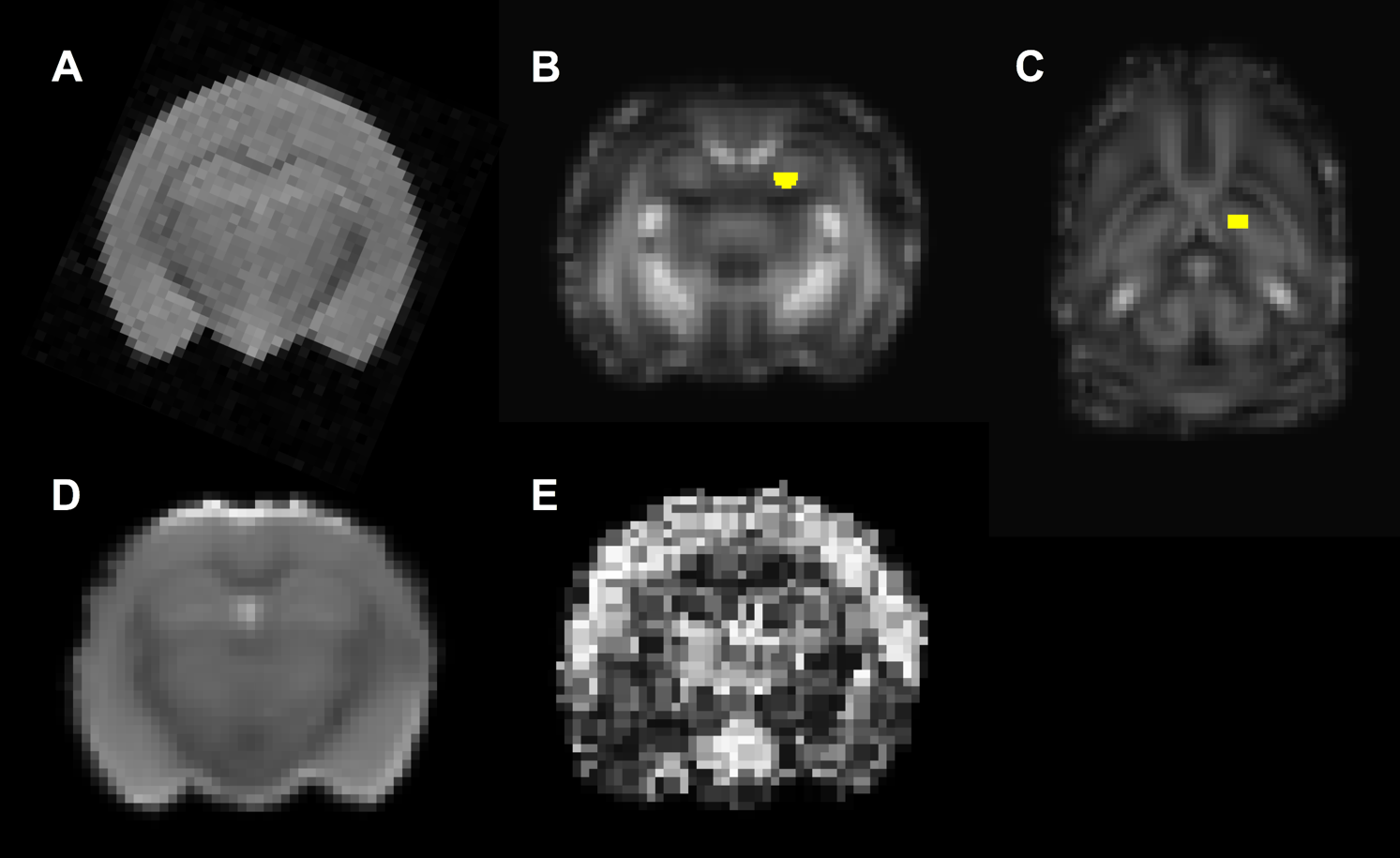
**

**Supplementary Figure 4:** (A) Coronal DWI, (B) coronal and (C) axial mean FA, (D) coronal MD, and (E) ODI maps of microglial depleted mouse brains 1 day following withdrawal of CSF1R inhibition. The yellow mask overlying the coronal and axial mean FA maps depicts the ROI overlaid on the left dentate gyrus.


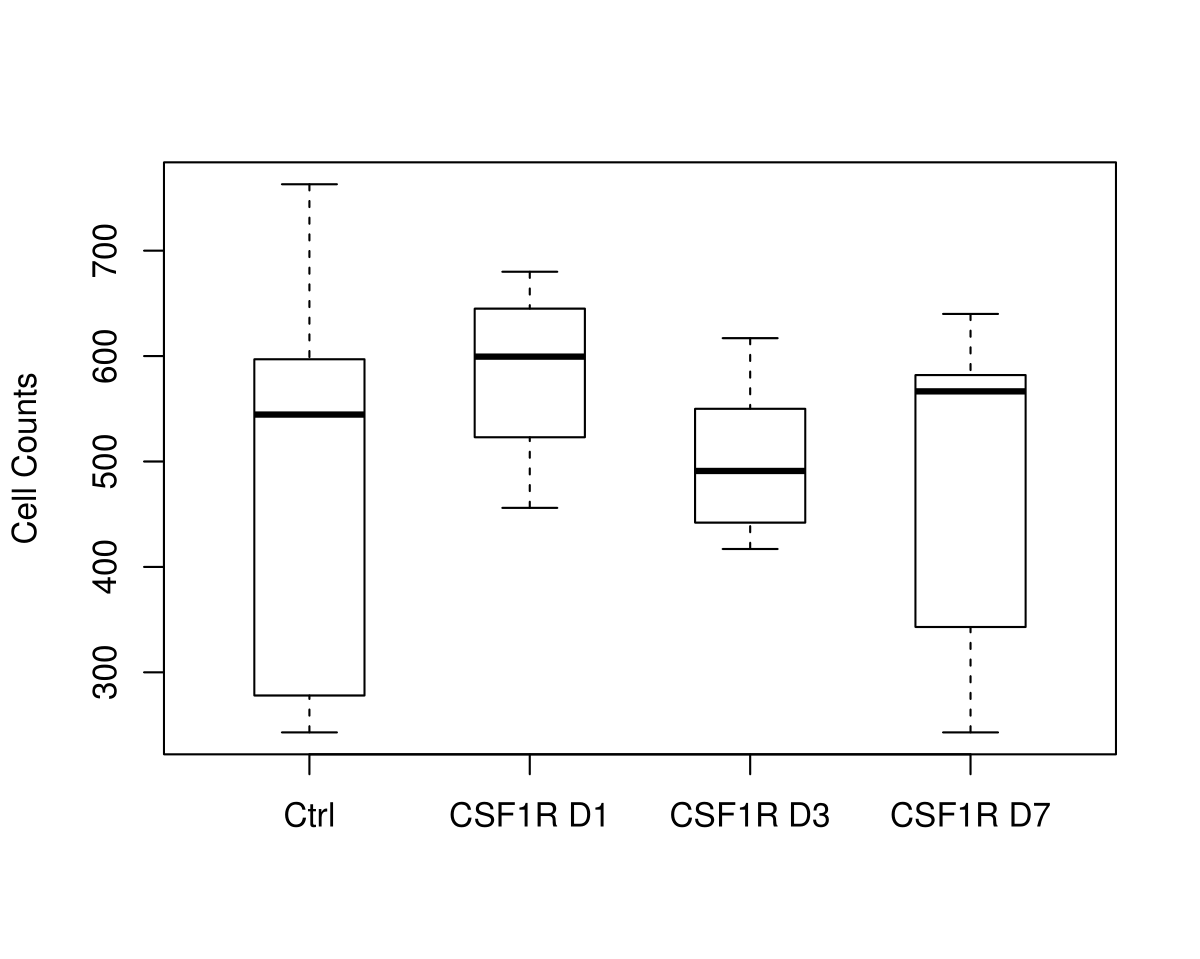
**Supplementary Figure 5:** Astrocyte counts can appear misleadingly high if there are no microglia to focus on during microscopy. The astrocyte counts on day 9 in Figure 5D seemed inappropriately high, albeit not significant upon initial evaluation. As there were no microglial to focus on, images had been taken with other cells in focus. In order detect any potential changes in astrocyte counts, all images were retaken while focused on the astrocytes. Particle analysis of these images clearly showed no significant change in astrocyte counts at *p<*0.05.
